# Letters to the Editor

**DOI:** 10.1590/1980-57642020dn14-010015

**Published:** 2020

**Authors:** Leonardo Ferreira Caixeta

**Affiliations:** Federal University of Goiás, Goiânia GO, Brazil

To the Editor:

We read carefully the case report by Borges et al.^1^ and disagree that the case presented by them has frontotemporal dementia as the main diagnosis for the following reasons:

1) Demographically, the patient is in an advanced age group which speaks against FTD, classically a form of presenile dementia,^2^ further suggesting the most common dementia at senile age, namely AD;
2) The symptoms presented are much more characteristic of AD than FTD, especially psychosis (persecutory delusions) and topographic disorientation, both of which are very rare in FTD yet very common in AD.^2^
3) The patient’s neuropsychological profile is much more consistent with AD because of the severe episodic memory and recognition deficits, cognitive functions usually preserved in FTD.
4) In terms of structural neuroimaging, her MRI is more characteristic of AD than FTD, since it discloses significant hippocampal atrophy (MTA=3), more markedly than frontal atrophy;^3^
5) In terms of functional neuroimaging, similarly, her SPECT is more characteristic of AD than FTD, with intense temporal and hippocampal hypoperfusion,^4^ especially in the left side.
6) The patient had no cholinergic side effects with galantamine, which is compatible with AD and incompatible with FTD (FTD patients have many severe gastrointestinal effects when exposed to anticholinesterasics).^5^

In addition to the above, it should be mentioned that the patient may not have responded to galantamine because the full therapeutic dose of the medication (24 mg/day) was not reached.

We suggest the most probable diagnosis in this case is the frontal variant of AD, or even the logopenic subtype of AD. Biomarkers would be useful to confirm this hypothesis, such as a CSF tau protein study.
