# Supplementary material for: Letters to the Editor
Source: Dement Neuropsychol. 2020 Jan-Mar;14(1):88–90. doi: 10.1590/1980-57642020dn14-010015 (PMC7077866; doi:10.1590/1980-57642020dn14-010015)
Supplement: Related texts

# AUTHORS’ REPLY

**Authors:** Saulo Queiroz Borges, Thiago Xavier Corrêa, Isabela Oliveira Azevedo Trindade, Rivadávio Fernandes Batista Amorim, Maria Alice de Vilhena Toledo

**Affiliations:** 1 MD, Universidade de Brasília (UnB), Hospital Universitário de Brasília (HUB), Brasília, DF, Brazil.; 2 Ft, MSc, Universidade de Brasília (UnB), Hospital Universitário de Brasília (HUB), Brasília, DF, Brazil.; 3 MSc, PhD, Universidade de Brasília (UnB), Hospital Universitário de Brasília (HUB).; 4 MD, MSc, PhD, Universidade de Brasília (UnB), Hospital Universitário de Brasília (HUB), Brasília, DF, Brazil.

**To the Editor:**

First of all, we would like to thank the esteemed reader for the comments that contributed to the deepening of the discussion about our case report.^1^ In fact, the case presented may raise questions about the diagnosis, and one of the differential diagnoses to be ruled out is Alzheimer’s Dementia (AD), especially its atypical forms. It is known that the diagnostic accuracy for a clinical diagnosis of dementias is limited, with sensitivity ranging from 71-88% and specificity ranging from 44-71%,^2^ with the definitive diagnosis still being made by anatomopathological examination. This diagnostic difficulty is even more challenging in the case of an elderly woman with a long history of major psychiatric disorder and a history of multiple crises and hospitalizations, with the potential for progressive cognitive decline. This fact leads to questioning even the diagnosis of dementia, since the most current clinical criteria require that this etiology be ruled out.^3^

However, some points may be better clarified in response to the comments made by the respected reader and colleague.

According to family members, our patient exhibited behavioral changes different from the mood episodes reported two years before the start of the follow-up at our service, at the age of 76. On this point, considering the Brazilian scenario, it is pertinent to highlight the frequent time difference between the onset of the first symptoms and the perception of those symptoms by companions, especially in patients with mental disorders, whose symptoms are, as a rule, attributed to the manifestation of the underlying psychiatric disease. It is also worth mentioning that, although FTD is characteristically a type of pre-senile dementia, onset of symptoms in FTD have been reported up to the ninth decade.^4^

The presence of a psychotic condition (paranoia) fails to indicate any specific subtype of dementia, since in FTDs, psychosis can be as common as in Alzheimer’s Dementia.^5^Although not detailed in the report, our patient had presented, in the initial evaluation, with partial impairment only in temporal orientation (2/5), being fully spatially oriented (5/5) on a screening exam (MMSE). It is known that dysfunctions in the prefrontal cortex circuit and in some of its connections (caudate nucleus, laterodorsal nucleus of the thalamus, and cingulate gyrus) seriously interfere with perception and temporal orientation, as well as with temporal synthesis and perception of duration, which may justify the impairment exhibited by our patient.^6^

Episodic memory and recognition are functions that are generally preserved in the early stages of FTD. However, at the time of her first evaluation, our patient already presented with significant impairment in different functional domains, thereby characterizing a moderate to advanced phase of a dementia syndrome (CDR 2), with the possibility of progression to other impairments besides executive dysfunction, classically affected in patients with FTD. It is worth mentioning that the patient described also had psychiatric comorbidity (bipolar disorder), which has the potential to lead to impairments in memory and verbal recognition, including to an euthymic state.^7^

Considering that the digitalized clipping of our patient’s neuroimaging data may not reveal all elements needed for a more accurate diagnosis, it is worth clarifying that all images were reviewed by two neuroradiologists from the university service, who identified global cerebral atrophy with frontal predominance. Furthermore, the presence of associated hippocampal atrophy is not a specific feature of Alzheimer’s disease, but rather supportive for its diagnosis, and which may be present in advanced stages of most cases of dementia syndromes.^8^

Our patient presented with moderate to severe hypometabolism/hypoperfusion in the frontal and temporal regions, which is one of the criteria for the diagnosis of probable FTD.^9^

After our patient started to take galantamine (8 mg with progression to 16 mg), she developed hyporexia and worse psychomotor agitation, a clinical picture described as possible side effects of anticholinesterases.^10^^,^ 11 There is even a concern with the use of acetylcholinesterase inhibitors in patients with bipolar 1 disorder, with some cases of manic shift described.^12^Thus, due to the risk of malnutrition worsening and uncontrolled bipolar disorder, we opted to suspend galantamine and adjust antipsychotics.

In the present case, unfortunately, CSF biomarker examination, which could have been used as an aid in the differential diagnosis, was not performed. However, the identification of a profile compatible with dementia in Alzheimer’s disease (low amyloid ß1-42 protein, and high TAU and p-TAU proteins) would not exclude the possibility of co-occurrence of FTD/AD neuropathologies^13^nor would it exclude the possibility (not ruled out) of neuroprogression in bipolar disorder.

## References

[1] Borges SQ, Correa TX, Trindade IOA, Amorim RFB, Toledo MAV (2019). Cognitive impairment in bipolar disorder: neuroprogression or
behavioral variant frontotemporal dementia?. Dement Neuropsychol.

[2] Beach TG, Monsell SE, Phillips LE, Kukull W (2012). Accuracy of the clinical diagnosis of Alzheimer disease at
National Institute on Aging Alzheimer Disease Centers,
2005-2010. J Neuropathol Exp Neurol.

[3] Frota NAF, Nitrini R, Damasceno BP, Forlenza O, Dias-Tosta E, da Silva AB (2011). Criteria for the diagnosis of Alzheimer’s
disease. Dement Neuropsychol.

[4] Kaplan H, Sadock B (2017). Comprehensive Textbook of Psychiatry.

[5] Thakur M, Blazer D, Steffens D (2014). Clinical Manual of Geriatric Psychiatry.

[6] Dalgalarrondo P (2019). Psicopatologia e Semiologia dos Transtornos Mentais.

[7] Goldberg JF, Chengappa KNR (2009). Identifying and treating cognitive impairment in bipolar
disorder. Bipolar Disord.

[8] Konijnenberg E, Fereshtehnejad SM, Kate MT, Eriksdotter M, Scheltens P, Johannsen P (2017). Early-Onset Dementia: Frequency, Diagnostic Procedures, and
Quality Indicators in Three European Tertiary Referral
Centers. Alzheimer Dis Assoc Disord.

[9] Cairns NJ, Bigio EH, Mackenzie IR, Neumann M, Lee VM, Hatanpaa KJ (2007). Neuropathologic diagnostic and nosologic criteria for
frontotemporal lobar degeneration: consensus of the Consortium for
Frontotemporal Lobar Degeneration. Acta Neuropathol.

[10] Birks J, Craig D (2006). Galantamine for vascular cognitive impairment. Cochrane database Syst Rev.

[11] Gill SS, Anderson GM, Fischer HD, Bell CM, Li P, Normand SL, Rochon PA (2009). Syncope and its consequences in patients with dementia
receiving cholinesterase inhibitors: a population-based cohort
study. Arch Intern Med.

[12] Ehrt U, Fritze F, Aarsland D (2011). Mania After Administration of Cholinesterase Inhibitors in
Patients With Dementia and Comorbid Bipolar Disorder. J Clin Psychopharmacol.

[13] Bang J, Spina S, Miller BL (2015). Frontotemporal dementia. Lancet (London, England).
